# The diagnostic value of BI-RADS grade 3 to 5 for breast masses is correlated with the expression of estrogen receptor, progesterone receptor, human epidermal growth factor receptor-2

**DOI:** 10.1097/MD.0000000000033208

**Published:** 2023-06-30

**Authors:** Tiejun He, Tiemei Shi, Wendong Luo, Yabo Ju, Ran Li

**Affiliations:** a Department of Ultrasound, The Third Affiliated Hospital of Jinzhou Medical University, Jinzhou, P.R. China; b Department of Ultrasound, Shengjing Hospital of China Medical University, Shenyang, P.R. China; c Department of General Surgery, The Third Affiliated Hospital of Jinzhou Medical University, Jinzhou, P.R. China; d Department of Obstetrics, The Third Affiliated Hospital of Jinzhou Medical University, Jinzhou, P.R. China; e Department of Anesthesiology, The Third Affiliated Hospital of Jinzhou Medical University, Jinzhou, P.R. China.

**Keywords:** breast imaging-reporting and data system, breast tumor, estrogen receptor, human epidermal growth factor receptor-2, progesterone receptor, ultrasound-guided puncture biopsy

## Abstract

The breast imaging-reporting and data system (BI-RADS) grading has a great advantage in diagnosing breast diseases, but with some limitations.

**Aims:**

The study analyzed the value of ultrasound-guided core needle biopsy (CNB) in diagnosing BI-RADS grades 3, 4, and 5 breast cancer.

**Methods:**

Breast cancer patients at BI-RADS grades 3 to 5 received breast ultrasonography, ultrasound-guided CNB and immunohistochemical examination. Receiver operating characteristic (ROC) curve was made to test diagnostic efficiency of regression model.

**Results:**

Calcification was positively correlated with expression of estrogen receptor (ER), progesterone receptor (PR) and human epidermal growth factor receptor (HER)-2. The areas of 4 ROC curves were 0.752, 0.805, 0.758, and 0.847, and the 95%CI was 0.660 to 0.844, 0.723 to 0.887, 0.667 to 0.849, and 0.776 to 0.918, respectively. BI-RADS grades 3 to 5 were positively correlated with expression of ER, PR and human epidermal growth factor receptor-2 (HER-2). Statistical significance existed between grade 5 and expression of ER, PR and HER-2, and between grade 4 and expression of HER-2.

**Conclusions:**

The study demonstrates that BI-RADS can be used as an effective evaluation method in the diagnosis of breast diseases before invasive operation, and it has higher diagnostic accuracy if combined with pathological examinations.

## 1. Introduction

Breast cancer is a common malignant tumor in women.^[1]^ In recent years, the incidence of breast cancer has been increasing year by year,^[1]^ ranking the first among all female malignant tumors in China.^[1]^ With the improvement of breast cancer treatments, the survival of breast cancer patients has been further increased.^[2]^ However, some patients at advanced stage or with poor molecular typing have poor prognosis, leading to deaths of the patients.^[2]^ With the continuous improvement of diagnosis and treatment, the screening of breast tumors has been significantly improved, and greatly improved the survival of breast cancer patients. The examination of breast tumors is mainly through combining clinical palpation with imaging examination. Ultrasonography is the most widely used method in clinical application. It usually judges benign and malignant tumors according to the shape, boundary, hyperechoic halo around lesions, echo type and blood supply.^[3,4]^ However, ultrasound examination still has its own limitations. In the examination of small breast cancer, the ultrasonographic signs are not obvious, leading to low detection rate and even missed diagnosis. The American College of Radiology has updated the new grading standard suitable for the Breast Imaging-Reporting and Data System (BI-RADS), providing a basis for the standardization and normalization of ultrasound diagnosis for breast lesions, which has enhanced the objectivity of ultrasound diagnosis of the disease. However, the application of BI-RADS grading in the diagnosis of atypical lesion or microlesions still has some limitations. Especially at grades 3 to 5, there are suspicious malignancies and misdiagnosis. For cases with atypical ultrasonographic signs, clinical manifestations should be considered, and puncture biopsy is needed.^[5]^ Core needle biopsy (CNB) is minimally invasive, simple and rapid for the sampling and diagnosis of suspicious lesions. It can avoid incision and enhance the cosmetic effect. Previous studies are mainly focused on how to improve the accuracy of lesions above grade 3 in BI-RADS classification using ultrasound technology.^[6–10]^ When necessary, hormone-dependent tumor markers such as estrogen receptor (ER), progesterone receptor (PR) and human epidermal growth factor receptor (HER)-2 are also considered.^[11]^ In addition, histological structure and morphological characteristics also determine the ultrasonographic manifestations of tumors, which are closely related to the occurrence, development, metastasis and recurrence of breast cancer.^[12]^

The aim of the present study is to retrospectively analyze the value of ultrasound-guided CNB in diagnosing BI-RADS grades 3, 4, and 5 breast cancer, and to study the correlation between ultrasonographic signs and molecular biological markers, in order to provide evidence for early diagnosis, treatment and prognosis evaluation of breast cancer.

## 2. Materials and methods

### 2.1. Subjects

A total of 120 patients who were diagnosed with breast cancer by ultrasound at our hospital between May 2016 and May 2019 were included in the study. The age range of the patients was 25 to 65 yeas, and the median age was 48 years. The diameter of nodules ranged between 5 mm and 25 mm. The inclusion criteria were: diagnosis of BI-RADS grades 3, 4, or 5 by high-frequency ultrasound and color Doppler ultrasound, as well as comparison with postoperative pathological results. All procedures performed in the current study were approved by the Ethics Committee of China Medical University. Written informed consent was obtained from all patients or their families.

### 2.2. BI-RADS diagnostic criteria

The diagnostic criteria^[13]^ were listed in Table 1. Ultrasound diagnostic criteria for grade 3 included: round or oval in shape; parallel with skin or width > height; clear borders; narrow and sharp edges; posterior echo enhancement or no change; no peripheral tissue changes; large calcification (>0.5 mm); no internal blood flow. Grade 3 was defined if round or oval in shape and parallel with skin or width > height were satisfied, and additional 3 or more criteria were met. Diagnostic criteria for grade 5 included: irregular shape; not parallel to skin or height > width; unclear boundary; strong echo halo sign; edges on both sides were not sharp, or irregular rear sound and shadow; change of surrounding tissues, interruption or disappearance of normal structure stratification, and/or skin thickening or depression; microcalcification (<0.5 mm); internal blood flow. Grade 5 was defined if 3 or more criteria were met. Grade 4 was defined if parallel with skin or width > height and clear borders of grade 3 or the criteria of grade 5 were not met. The ultrasonographic manifestations of lesions under BI-RADS grading were shown in Table 2.

**Table 1:** BI-RADS diagnostic criteria.

Grades	Diagnostic criteria	Other information
0	Examination was incomplete or unsatisfactory	Imaging examinations are required.
1	Negative lesions	No lesions were observed and routine follow-ups were needed.
2	Benign lesions	No malignant signs were observed, and only simple cyst or fibroadenoma without obvious change were observed. Routine follow-up was needed.
3	Possibility of benignity	The likelihood of breast malignancies is low, and asymptomatic fibroadenomas or complicated cysts were observed. Follow-up every 6 mo.
4	Suspicious malignant lesions	Cancerous tumor is possible, but needs to be confirmed by biopsy.
5	Malignancy possibility extremely high	Confirmation by surgery or puncture biopsy was needed.
6	Definite malignant pathological results	Corresponding measures were needed

BI-RADS = breast imaging-reporting and data system.

**Table 2:** Ultrasonographic manifestations of lesions under BI-RADS grading (n = 120).

Ultrasonographic manifestations		BI-RADS grading		
		3	4	5
Mass morphology	Regular	15	15	11
	Irregular	6	27	46
Boundary blur	No	18	11	5
	Yes	3	31	52
Mass size	≤2 cm	14	30	12
	>2 cm	7	12	45
Calcification in mass	No	9	22	6
	Yes	11	20	51
Subaxillary lymphadenopathy	No	13	7	10
	Yes	8	35	47
CDFI	Not abundant	16	17	3
	Abundant	5	25	54

BI-RADS = breast imaging-reporting and data system, CDFI = color Doppler flow imaging.

### 2.3. Ultrasonography

**Figure 1.**
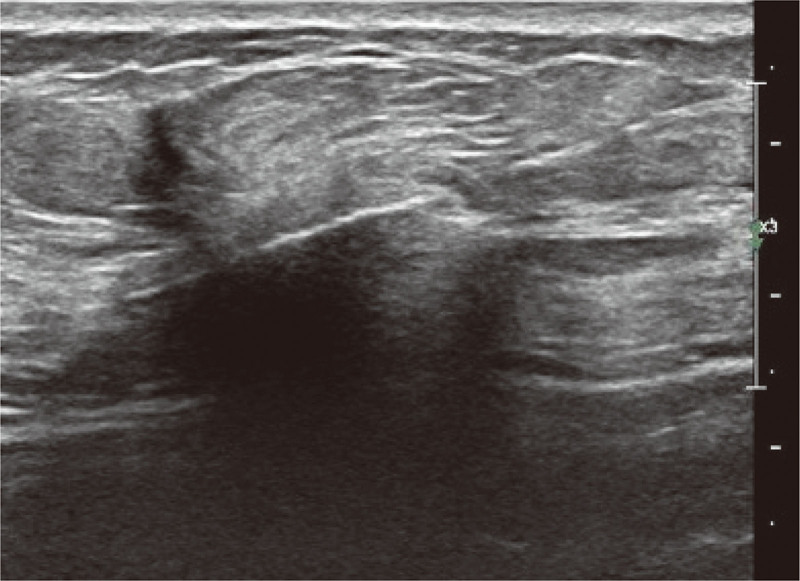
Ultrasound-guided puncture biopsy of breast tumors. CNB was performed under the guidance by Color Ultrasound Diagnostic Instrument (GE Healthcare, Boston, MA, USA). The frequency of the probe was 10 MHz. Magnum Disposable Core Tissue Biopsy Needle (Bard; BD Biosciences, Franklin Lakes, NJ, USA) had a needle groove length of 20 to 22 mm. CNB = core needle biopsy.

**Figure 2.**
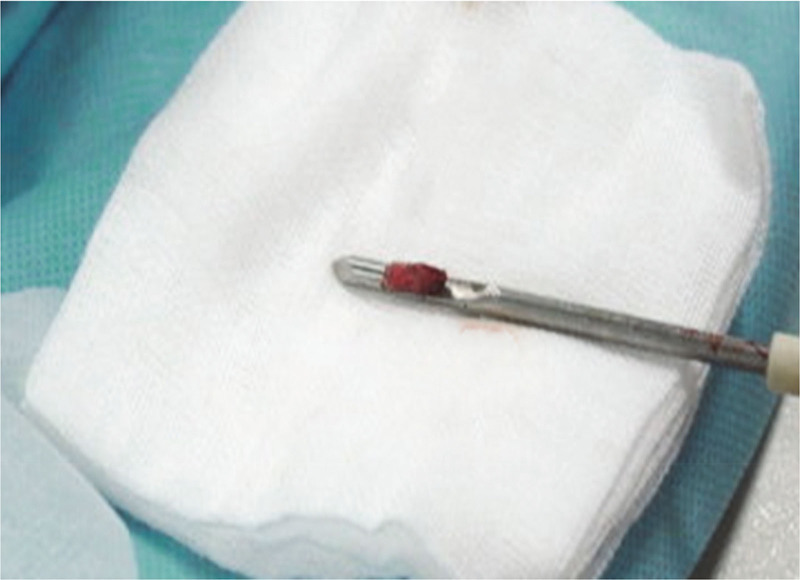
Pathological tissue in the needle of puncture biopsy. Breast masses of BI-RADS grade 3 to 5 were selected for the study. The selection of puncture points should be maintained within the surgical reach. The distance between puncture point and pleura should be greater than the range of puncture needle. The insertion depth of biopsy needle should be determined according to the depth, size and correlation of the lesions. General operations retained 3 to 6 samples. After operation, the biopsy needle was pulled out. BI-RADS = breast imaging-reporting and data system.

CNB was performed under the guidance by Color Ultrasound Diagnostic Instrument (GE Healthcare, Boston, MA). The frequency of the probe was 10 MHz. Magnum Disposable Core Tissue Biopsy Needle (Bard; BD Biosciences, Franklin Lakes, NJ) had a needle groove length of 20 to 22 mm. During color Doppler ultrasonography of the breast, the details of the lesions were observed and recorded, and the distance between the mass and the nipple was measured. BI-RADS grading was performed according to the shape, margin, direction, internal echo, posterior echo, changes of surrounding tissues, calcification, blood supply and lymphatic drainage. Breast masses of BI-RADS grade 3 to 5 were selected for the study. The selection of puncture points should be maintained within the surgical reach. The distance between puncture point and pleura should be greater than the range of puncture needle. The insertion depth of biopsy needle should be determined according to the depth, size and correlation of the lesions. General operations retained 3 to 6 samples. After operation, the biopsy needle was pulled out, the size of the tissue strip was recorded and the tissues were placed in fixative fluid for subsequent pathological examination (Figs. 1 and 2).

### 2.4. Pathological examinations

Breast puncture specimens were fixed with 10% neutral formalin solution, embedded with paraffin, sliced into 4 μm slices, and stained with hematoxylin and eosin. Then, histopathological diagnosis was performed. Expression of ER, PR and human epidermal growth factor receptor-2 (HER-2) was determined by immunohistochemistry according to the manufacturer manual (EnVision Detection Systems; Agilent, Santa Clara, CA).

### 2.5. Judgement of immunohistochemical results

Positive results of ER and PR were judged by the following 2 scoring systems. First, ten high-power visual fields of uniformly colored tumors were selected, the number of positive cells and the total number of cells were counted, and the percentage of positive cells was calculated. If no positive cells were observed, 0 score was recorded. If the percentage of positive cells was within 1% to 10%, 1 score was recorded. If the percentage of positive cells was within 11% to 50%, 2 scores were recorded. If the percentage of positive cells was within 51% to 80%, 3 scores were recorded. If the percentage of positive cells was >80%, 4 scores were recorded. The second scoring system depended on coloring depth. If no color was observed, 0 score was recorded. If light yellow was observed, 1 score was recorded. If light brown was observed, 2 scores were recorded. If dark brown was observed, 3 scores were recorded. The scores in the 2 systems were added up. If the total score was 0 or 1, negative result (−) was achieved. If the total score was 2, weak positive result (+) was achieved. If the total score was 3 to 4, positive result (++) was achieved. If the total score was 5 to 6, strong positive result (+++) was achieved (Table 3).

**Table 3:** Positive results of each marker and positive controls.

Biological markers	Staining sites	Positive controls
ER	The nuclei of the tumor cells were clear brown-yellow.	Peripheral normal ductal epithelial cells of breast
PR	The nuclei of the tumor cells were clear brown-yellow.	Peripheral normal ductal epithelial cells of breast
HER-2	Tumor cell membrane was brown yellow	Known positive infiltrating breast cancer cells

ER = estrogen receptor, HER-2 = human epidermal growth factor receptor-2, PR = progesterone receptor.

Positive result of HER-2 was judged by membrane coloring intensity and the proportion of colored cancer cells. If no coloring was observed and <1% cancer cells were stained, the score was 0+. If the coloring was weak and <10% cancer cells were stained, the score was 1+. If the coloring was clear and more than 10% cancer cells were stained, the score was 2+. If the coloring was strong and more than 10% cancer cells were stained, the score was 3 + (Table 3).

### 2.6. Statistical analysis

The results were analyzed using SPSS 20.0 statistical software (IBM, Armonk, NY). Chi-square test was used to compare the counting data between groups. Spearman grade correlation analysis was used to analyze the correlation between indicators. *P* < .05 indicated significant difference between groups. The detection rate, specificity, sensitivity, missed diagnosis rate and misdiagnosis rate of the corresponding examination methods were calculated. Receiver Operating Characteristic (ROC) curve was drawn to test the diagnostic efficiency of regression model.

## 3. Results

### 3.1. Comparisons of puncture biopsy and postoperative pathological results among patients with single breast nodule at BI-RADS stages 3, 4, and 5

For patients at BI-RADS grades 3, 4, and 5, the specificity of breast cancer diagnosis was 100% for all, the sensitivity was 70.0%, 89.2%, and 80.9%, respectively. The false negative rates were 30.0%, 10.7%, and 19.1%, respectively. The accuracy was 90.4%, 92.8%, and 85.9%, respectively. Specificity, sensitivity, accuracy and false negative rates in the diagnosis of nodular lesions were significantly different among patients at grades 3, 4, and 5 (*P* < .05) (Table 4).

**Table 4:** Comparisons of puncture biopsy and postoperative pathological results among patients with single breast nodule at BI-RADS stages 3, 4, and 5.

BI-RADS grades	Specificity (%)	Sensitivity (%)	Accuracy (%)	False negative rate (%)
3	100	70.0	90.4	30.0
4	100	89.2	92.8	10.7
5	100	80.9	85.9	19.1
χ^2^	9.851	6.777	10.524	41.103
P	0.007	0.034	0.005	0.000

BI-RADS = breast imaging-reporting and data system.

*P* < .05 indicates significant difference.

### 3.2. Correlation between ultrasound signs of breast masses and expression of ER, PR and HER-2

The mass shape, size, boundary burr, calcification, lymph node enlargement and color Doppler flow imaging (CDFI) in ultrasound signs were positively correlated with biological indicators. Boundary burr, mass shape, mass size, lymph node enlargement and CDFI expression were not significantly different between groups for ER, PR and HER-2 (*P* > .05). Microcalcification was positively correlated with positive expression of ER, PR and HER-2 (*P* < .05) (Table 5).

**Table 5:** Correlation between ultrasound signs of breast masses and expression of ER, PR, and HER-2.

Ultrasound signs	Biological indicators	No. of cases	ER			PR			HER-2		
			Positive	χ^2^	*P*	Positive	χ^2^	*P*	Positive	χ^2^	*P*
Mass shape	Regular	41	29	0.296	.370	30	0.111	.451	20	1.256	.177
	Irregular	79	52			60			47		
Boundary burr	No	34	12	2.444	.076	14	2.745	.064	18	0.003	.333
	Yes	86	54			56			40		
Mass size	≤2 cm	56	43	0.771	.251	47	0.013	.219	29	0.698	.258
	>2 cm	64	46			49			38		
Calcification	No	37	19	10.48	.002	24	7.169	.008	15	6.292	.011
	Yes	52	43			46			35		
Subaxillary lymphadenopathy	No	30	17	0.979	.220	14	0.714	.263	10	2.517	.084
	Yes	90	60			65			45		
CDFI	Not abundant	36	19	0.869	.232	24	2.358	.098	10	0.057	.484
	Abundant	84	52			67			40		

CDFI = color Doppler flow imaging, ER = estrogen receptor, HER-2 = human epidermal growth factor receptor-2, PR = progesterone receptor.

### 3.3. Multiparameter regression model has certain clinical significance in the diagnosis of malignant breast tumors

**Figure 3.**
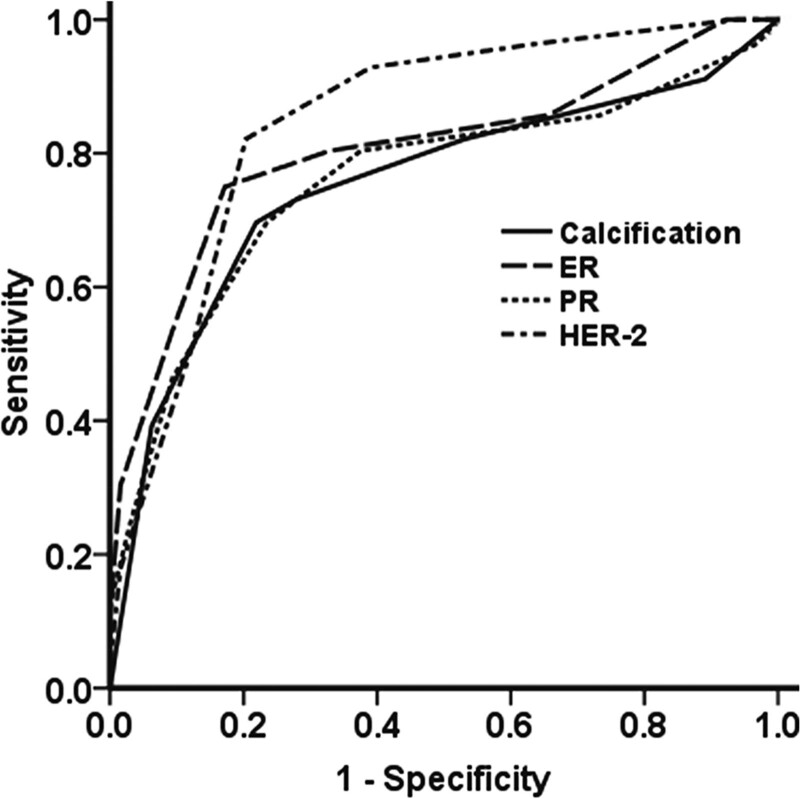
ROC curves of ER, PR, and HER-2 multiparameter models. ROC curves for multiparameter regression models of microcalcification, ER, PR, and HER-2 were drawn. The area under the curve (AUC) for the 4 ROC curves were 0.752, 0.805, 0.758, and 0.847, respectively. Their 95% CI were 0.660 to 0.844, 0.723 to 0.887, 0.667 to 0.849, and 0.776 to 0.918, respectively. The ROC shapes and AUC show that multiparameter regression model has certain clinical significance in the diagnosis of malignant breast tumors. ER = estrogen receptor, HER-2 = human epidermal growth factor receptor-2, PR = progesterone receptor, ROC = receiver operating characteristic.

ROC curves for multiparameter regression models of microcalcification, ER, PR and HER-2 were drawn. The area under the curve for the 4 ROC curves were 0.752, 0.805, 0.758, and 0.847, respectively. Their 95% CI were 0.660 to 0.844, 0.723 to 0.887, 0.667 to 0.849, and 0.776 to 0.918, respectively (Fig. 3). The ROC shapes and area under the curve show that multiparameter regression model has certain clinical significance in the diagnosis of malignant breast tumors.

### 3.4. Correlation between BI-RADS grades and expression of ER, PR, and HER-2

According to Spearman rank correlation test, different BI-RADS grades were positively correlated with expression of ER, PR, and HER-2. In BI-RADS grade 3, there was no statistical significance for the expression of ER, PR and HER-2 (*P* > .5). In BI-RADS grade 5, there was statistical significance for the expression of ER, PR and HER-2 (*P* < .5). In BI-RADS grade 4, there was no statistical significance for the expression of ER and PR (*P* > .5) (Table 6).

**Table 6:** Correlation between BI-RADS grades and expression of ER, PR, and HER-2.

Grades		ER	PR	HER-2
3	r	0.050	0.048	0.168
	P	0.416	0.418	0.232
4	r	0.017	0.255	0.292
	P	0.457	0.051	0.030
5	r	0.352	0.402	0.348
	P	0.003	0.001	0.004

BI-RADS = breast imaging-reporting and data system, ER = estrogen receptor, HER-2 = human epidermal growth factor receptor-2, PR = progesterone receptor.

## 4. Discussion

### 4.1. Breast cancer management demands improvements in diagnostics and treatment

In recent years, the incidence of breast cancer has shown an increase trend, and the age of onset is decreasing.^[14]^ Early detection, accurate diagnosis and timely treatment of breast cancer directly affect the treatment effect and prognosis.^[15,16]^ Ultrasound-guided puncture is convenient, minimally invasive and easy to be widely used in clinic. It has become the preferred examination method for breast diseases. In the 2017 edition of Guidelines and Specifications for the Diagnosis and Treatment of Breast Cancer,^[13]^ the Chinese Anti-Cancer Association recommends performing preoperative image-guided biopsy in qualified hospitals. The pathological and histological morphology of tumors is the basis of the ultrasonographic manifestations of breast cancer. Understanding this is essential for analyzing the ultrasonographic features of breast cancer. Mammary gland is a hormone-dependent organ. The expression of hormone receptors is often altered in the occurrence of breast cancer. Therefore, breast cancer is also considered as a hormone-dependent tumor. At present, there are more and more mature studies in breast cancer, and the molecular biological indicators widely used in clinic include ER, PR, and HER-2, which are closely related to the occurrence, development, diagnosis, treatment and prognosis of breast cancer. They are the most common biomarkers indicating the biological behavior and prognosis of breast cancer.

### 4.2. The advantages of the diagnosis method proposed in this study

The diagnostic method introduced here is based on the analysis of biological factors related to histological samples obtained from ultrasound images and biopsy. The higher the relevant grading index of BI-RADS grading system is, the worse the prognosis is. They are complementary to biological functions. Different grading and biological indexes can evaluate the development and progress of the tumor.^[17]^ Because the BI-RADS grading system is obtained through the relevant indicators of ultrasound images, and through the creation of risk assessment model with biomarkers, the risk assessment model has a certain correlation with the tumor progression before the clinical manifestation of the disease, which is particularly valuable in clinical application.

### 4.3. Presented approach demonstrates a potential to improve breast cancer management

For patients with BI-RADS grades 3 to 5, the specificity in the diagnosis of breast lesions was 100%, and the sensitivities were 70.0%, 89.2%, and 80.9%, respectively. There was a positive correlation among the groups, with statistical significance. The false negative rate of CNB for patients at BI-RADS grade 3 was 30%, which was significantly higher than those of patients at BI-RADS grade 4 or 5, being acceptable according to a previous report.^[18]^ The main reasons are the number of puncture samples, the accuracy of sampling and the pathological types of lesions. Puncture biopsy is mainly towards cystic tissues, and the solid part is usually little, so it is easy to misdiagnose highly differentiated and low-grade malignant breast cancer as benign nodules. Therefore, it is suggested that an accuracy of 99% can be achieved by taking at least 4 to 5 samples during CNB, while other researchers believe that multi-point puncture has no significant effect on diagnosis.^[7–10]^ It is also believed that false negative rate is different among different pathological types. For example, ultrasound findings of in situ and highly differentiated carcinomas are atypical and classified as BI-RADS grade 3, resulting in missed diagnosis. In the meantime, some special types of benign lesions are classified as BI-RADS grade 5 because of malignant signs. This requires that we strictly follow the BI-RADS classification criteria, and observe the dynamic state of each section layer by layer.

### 4.4. Relationship between biological indexes and ultrasonic characteristics

Ultrasound signs are closely related to pathological basis. The shape, boundary and size of tumors are very similar to those of pathological specimens. The growth patterns of breast cancer in tissue structure are different. In this study, the shape, size, marginal burr, calcification, lymph node enlargement and CDFI observed in ultrasound examination were positively correlated with the biological manifestations. Significant differences were only found between calcification and the positive expression of ER, PR and HER-2. Up to now, the mechanism of calcification in breast cancer remains unclear. However, it is reported that bone matrix proteins are found in breast cancer tissues, and calcification in calcium and phosphorus deposition environment formed by bone matrix protein metabolism is associated with positive expression of ER, PR, and HER-2.^[19,20]^ The amount and size of calcification are closely related to the malignant degree of breast cancer and can be used as an important characteristic index for the diagnosis of breast cancer. In the present study, the expression of ER, PR, and HER-2 in calcification group was significantly higher than that in non-calcification group, being consistent with the above conclusions. A study shows that the diagnostic efficiency of lymph node biopsy for breast cancer is high, and the area under ROC is higher than 0.9.^[20]^. The diagnostic efficiency of BI-RADS itself in breast masses with calcification, solid, cystic and cystic solid lesions is relatively lower than that of puncture biopsy, and the misdiagnosis rate is high due to the nature of the lesions. In the process of puncture, if the breast mass is cancerous with cystic lesions, the measured biomarker indexes are low or false negative. The combination of the 2 is able to diagnose breast lesions and help clinicians understand the correlation between ultrasound findings and pathology, so as to make a diagnostic model for clinicians before follow-up treatment.

### 4.5. Diagnostic values of biological indexes and some ultrasonic characteristics

In the present study, the results of pathological examination after breast puncture biopsy and pathological diagnosis of the specimens after operation were taken as the gold standard. The areas under the 4 ROC curves was 0.752, 0.805, 0.758, and 0.847, respectively, and the 95% CIs were 0.660 to 0.844, 0.723 to 0.887, 0.667 to 0.849, and 0.776 to 0.918, respectively. Therefore, the area under ROC curve has certain clinical significance in the diagnosis of malignant breast tumors. Different areas under ROC between lesion biopsy and lymph node biopsy might be due to their different ultrasound shapes. Lymph node enlargement or metastasis is usually discovered at stages higher than grade 5 in BI-RADS classification. Therefore, effective judgment of BI-RADS classification, combined with biological manifestations, can effectively improve the diagnostic efficiency. At present, there are few studies on the biological expression of breast cancer at BI-RADS grades 3 to 5, and many studies have focused on a single ultrasound indicator.^[21,22]^ In the present study, the expression levels of ER, PR and HER-2 were positively correlated with BI-RADS grades 3, 4 and 5. However, no statistical significance existed between grade 3 and the expression of ER, PR and HER-2. Statistical significance existed between grade 5 and the expression of ER, PR and HER-2. In addition, no statistical significance existed between grade 4 and the expression of ER and PR, while statistical significance existed between grade 4 and the expression of HER-2. When breast cancer occurs, cells containing ER, PR and HER-2 proliferate malignantly, causing positive expression of ER, PR and HER-2. This is obviously related to the changes among grades 3 to 5 in BI-RADS classification. For BI-RADS grade 4, the expression of ER, PR and HER-2 were not consistent, possibly because, with the increase of BI-RADS grades, HER-2 amplification and expression can inhibit cell apoptosis, regulate cell differentiation, promote cell proliferation^[23]^ and neovascularization, leading to breast cancer.

## 5. Conclusions

The present study demonstrates that ultrasound-guided CNB is easy to operate and can cause weaker trauma, and thus has high diagnostic value for breast cancer in combination with BI-RADS classification. BI-RADS classification is accurate, but there is still a certain rate of missed diagnosis. The combination of breast ultrasound signs with BI-RADS classification can predict the biological indicators of breast cancer, such as ER, PR and HER-2. The presented achievements may improve the overall BC management by providing evidence for early diagnosis, treatment and prognosis evaluation of breast cancer. Although many tumors have been effectively treated, the exact mechanisms of tumor occurrence and development still remain unclear. Cancer is a complex systemic disease, involving various abnormalities of genes, proteins, metabolites and medical images.^[24]^ In particular, predictive biomarkers for treatment stratification, diagnostic biomarkers for early detection and prognostic biomarkers for estimating the clinical outcome of patients are essential for the prevention of tumors. The pathogenesis of breast cancer should be studied from gene mutation, abnormal glycosylation level of protein, metabolism and imaging manifestations.

## Acknowledgments

The authors wish to thank their department and research team for their help and dedication.

## Author contributions

**Conceptualization:** Tiejun He.

**Data curation:** Tiemei Shi, Yabo Ju.

**Formal analysis:** Tiejun He, Wendong Luo, Yabo Ju.

**Funding acquisition:** Tiejun He.

**Investigation:** Tiemei Shi, Wendong Luo, Yabo Ju.

**Methodology:** Tiemei Shi.

**Project administration:** Tiejun He, Wendong Luo, Ran Li.

**Resources:** Tiemei Shi, Ran Li.

**Software:** Tiejun He, Yabo Ju.

**Supervision:** Tiemei Shi, Yabo Ju, Ran Li.

**Validation:** Tiejun He, Wendong Luo, Ran Li.

**Visualization:** Tiemei Shi, Ran Li.

**Writing – review & editing:** Tiejun He.

**Writing – original draft:** Tiemei Shi.
